# Physical and Mental Health Impacts of the COVID-19 Pandemic among US Adults with Chronic Respiratory Conditions

**DOI:** 10.3390/jcm10173981

**Published:** 2021-09-02

**Authors:** Lulu Wei, Jessica Y. Islam, Eduardo A. Mascareno, Argelis Rivera, Denise C. Vidot, Marlene Camacho-Rivera

**Affiliations:** 1 Department of Epidemiology, SUNY Downstate Health Sciences University, New York, NY 11203, USA; lulu.wei@downstate.edu; 2 Cancer Epidemiology Program, H. Lee Moffitt Cancer Center and Research Institute, Tampa, FL 33612, USA; jessica.islam@moffitt.org; 3 Department of Public Health, SUNY College at Old Westbury, New York, NY 11568, USA; emascare@oldwestbury.edu; 4 Department of Medicine, Mount Sinai Morningside Hospital, New York, NY 10025, USA; argelis.rivera@mountsinai.org; 5 School of Nursing and Health Studies, University of Miami, Coral Gables, FL 33146, USA; dvidot@med.miami.edu; 6 Department of Community Health Sciences, SUNY Downstate Health Sciences University, New York, NY 11203, USA

**Keywords:** respiratory conditions, COVID-19, SARS-CoV-2, mental health, depression, anxiety

## Abstract

Adults living with chronic respiratory diseases are at higher risk of death due to COVID-19. Our objective was to evaluate the physical and mental health symptoms among US adults living with chronic respiratory conditions. We used data of 10,760 US adults from the nationally representative COVID-19 Impact Survey. Chronic respiratory conditions were self-reported and included asthma (14.7%), chronic obstructive pulmonary disease or COPD (4.7%), and bronchitis/emphysema (11.6%). We used multivariable Poisson regression to evaluate physical health symptoms. We estimated associations of mental health symptoms using multinomial logistic regression. In multivariable models, adults with asthma were more likely to report physical symptoms including runny or stuffy nose, chest congestion, fever, and chills. In addition, adults with COPD were more likely to report several physical symptoms including fever (adjusted prevalence ratio [aPR]: 1.37, 95% confidence interval [CI]: 1.09–1.72), chills (aPR: 2.10, 95% CI: 1.67–2.64), runny or stuffy nose (aPR: 1.78, 95% CI: 1.39–2.27), chest congestion (aPR: 2.14, 95% CI: 1.74–2.61), sneezing (aPR: 1.59, 95% CI: 1.23–2.05), and muscle or body aches (aPR: 1.38, 95% CI: 1.06–1.81). Adults with chronic respiratory conditions are more likely to report physical and mental health symptoms during the COVID-19 pandemic compared to others. Providers should prioritize discussing mental health symptom management as the pandemic continues to be a public health concern in the US.

## 1. Introduction

COVID-19, caused by severe acute respiratory syndrome coronavirus 2 (SARS-CoV-2) infection, has resulted in over 600,000 deaths in the United States since 20 January 2020, the date of the first confirmed case in the US [1,2]. Currently, the United States has the highest rate of COVID-19-related deaths per 100,000 people in the world. To combat the spread of SARS-CoV-2, the Centers for Disease Control and Prevention (CDC) has promoted uptake and maintenance of preventative behaviors such as hand washing, 6-feet social distancing, utilizing telemedicine, and avoiding non-essential visits to doctor’s offices. Adherence to these recommendations may pose a challenge among individuals with chronic respiratory conditions given their increased health care needs and utilization to manage their health compared to the general population [3].

Simultaneously, the CDC has identified patients with preexisting conditions such as asthma, chronic obstructive pulmonary disease (COPD), chronic bronchitis, and emphysema to be vulnerable populations for COVID-19 incidence and mortality [4,5,6]. The US has a significant COPD and asthma burden: in 2014–2015, over 15.9 million or 5.9% of US adults were living with COPD including emphysema and chronic bronchitis, and in 2018, over 19.2 million or 7.7% of US adults were living with asthma [7,8]. Multiple meta-analyses have demonstrated that respiratory comorbidities are associated with more severe COVID-19 symptoms and/or higher mortality [4,9]. This disparate burden may be related to higher angiotensin-converting enzyme 2 receptor expression in individuals with COPD and asthma [10]. The evidence that those with pre-existing respiratory conditions may have worse COVID-19 prognoses is growing [11,12]. 

Challenges exist in the identification and treatment of COVID-19 among individuals with pre-existing respiratory conditions due to overlap between COVID-19 symptoms and their existing chronic conditions. Symptoms of early COVID-19 infection may include fever, fatigue, myalgia, headache, anosmia, ageusia, sore throat, nausea, and diarrhea [10]. Respiratory symptoms of COPD, which are also common among individuals with COVID-19, include cough, sputum production, dyspnea, and fatigue, overlapping with those of COVID-19 [10]. Common symptoms may obscure a patient or physician’s suspicions of infection [13]. Proper symptom management of chronic pulmonary conditions can make clinical diagnosis easier and can decrease the number of hospital visits for acute exacerbations [13].

Beyond the physical consequences of COVID-19 exposure, the COVID-19 pandemic has led to an increase in mental health-related conditions among US adults due to recommended preventive behaviors such as social distancing and quarantining [14,15,16]. To our knowledge, no prior work has described the mental health of adults living with chronic respiratory conditions across the US during the COVID-19 pandemic. Prior studies before the onset of the pandemic demonstrate that adults living with COPD and asthma also report increased experiences of anxiety (e.g., generalized anxiety disorder, social phobia, and panic disorder) and depressive symptoms (e.g., major depression and dysthymia). Patients living with these comorbid conditions experience greater symptom burden and are more likely to utilize health services, which may in turn put them at greater risk for COVID-19 morbidity [17,18]. Quantifying mental distress among these COVID-19-vulnerable populations may aid in developing targeted interventions to improve their physical and mental health, and reduce their risk of developing COVID-19. However, population-based data on the physical and mental consequences of the COVID-19 pandemic among COPD and asthma patients are lacking. 

Our objective was to evaluate the physical and mental health symptoms reported during the COVID-19 pandemic among a nationally representative sample of US adults, comparing those with and without chronic respiratory conditions. Understanding the physical and mental health burden amongst patients with chronic COPD and asthma may improve patient self-management and help patients understand when to utilize in-person health services.

## 2. Materials and Methods

### 2.1. COVID-19 Impact Survey

This analysis was conducted using the COVID-19 Household Impact Survey Data gathered from the AmeriSpeak^®^ panel [19]. The survey and panel were conducted and created by NORC at the University of Chicago for the Data Foundation to capture information about the physical health, mental health, habits, and demographics of US residents. Data from Week 1 (20–26 April 2020), Week 2 (4–10 May 2020), and Week 3 (30th May–8th June 2020) are publicly available and were merged for this analysis. The analytic sample of 10,760 adults was weighted according to data from the 2020 Current Population Survey conducted by the US Census Bureau and the US Bureau of Labor Statistics to create a US-representative sample of adults aged ≥18 years. The number of participants invited and the percentage of interviews completed by week are as follows: 11,133 invited with 19.7% interviews completed during Week 1; 8570 invited with 26.1% interviews completed (Week 2); and 10, 373 invited with 19.7% interviews completed (Week 3).

NORC of the University of Chicago created the AmeriSpeak^®®^ panel using NORC’s National Sample Frame area probability sampling and address-based sampling. This sampling method allowed the AmeriSpeak panel to be representative of 97% of US households, apart from those with unlisted USPS Delivery Sequence File addresses, P.O. Box addresses, and newly constructed residences. Surveys were completed in English or Spanish online or by telephone with a NORC-trained interviewer. Respondents were eligible for a $5 monetary incentive in exchange for participating. Only one panelist per household was eligible to respond.

### 2.2. Statistical Analysis

Our primary outcomes for this analysis were physical and mental health symptoms self-reported in the last 7 days. To evaluate physical symptoms, we used participants’ responses (yes/no) to the following question: “Have you experienced any of the following symptoms in the past 7 days or not?” Of the 17 options, participants were able to select all that apply: fever, chills, runny or stuffy nose, chest congestion, skin rash, cough, sore throat, sneezing, muscle or body aches, headaches, fatigue or tiredness, shortness of breath, abdominal discomfort, nausea or vomiting, diarrhea, changed or loss sense of taste or smell, and loss of appetite.

To evaluate mental health symptoms, we used participants’ responses to the following questions: “In the past 7 days, how often have you: felt nervous, anxious, or on edge; felt depressed; felt lonely; felt hopeless about the future; and had physical reactions such as sweating, trouble breathing, nausea, or a pounding heart when thinking about your experience with the coronavirus pandemic.” Participants were able to choose from the following list of options for each mental health symptom: not at all or less than 1 day, 1–2 days, 3–4 days, and 5–7 days.

The primary predictor for this analysis was participants’ self-report of a chronic respiratory condition. Participants were then asked to reply “yes, no, or not sure” to the following question: “Has a doctor or other health care provider ever told you that you have any of the following: diabetes; high blood pressure or hypertension; heart disease, heart attack or stroke; asthma; chronic lung disease or COPD; bronchitis or emphysema; allergies; a mental health condition; cystic fibrosis; liver disease or end-stage liver disease; cancer; a compromised immune system; or overweight or obesity.”

The following covariates were included a priori in the multivariable analyses based on a review of the scientific literature: age categories (18–29, 30–44, 25–59, 60+), sex (male and female), race/ethnicity categories (non-Hispanic White, non-Hispanic Black, Hispanic, non-Hispanic Other), insurance status (yes/no), obesity (obese and non-obese), and history of allergies (yes/no).

We used chi-square tests to compare physical and mental health symptoms among adults with pre-existing respiratory conditions compared to the general US adult population. Poisson regression was used to calculate the prevalence ratios and 95% confidence intervals associated with reporting “yes” to COVID-19-related physical symptoms (versus no) by respiratory disease history. The multivariable logistic regression models included adjustments for age, sex, race/ethnicity, insurance status, obesity, and allergy history.

Next, we used multinomial logistic regression to compare mental health symptoms reported in the last 7 days among US adults with pulmonary conditions to those without adjustment for age, sex, race/ethnicity, insurance status, obesity, and allergy history. We conducted a sensitivity analysis to parse out those without a history of a mental health condition through the following question: “Have you ever been diagnosed by a doctor or has a health care provider ever said you have a mental health condition?” Although “mental health condition” is a broad statement, we were able to focus our analysis on those without clinical depression and anxiety using this approach. Based on the exploratory nature of this analysis, we did not include an adjustment for multiple comparisons. All statistical analyses were conducted using Stata IC 15 (StataCorp LLC, College Station, TX, US). Sampling weights were applied to provide results that were nationally representative of the US adult population.

## 3. Results

Of the 10,760 respondents, 54.1% of the study population was 45 years of age and above, 51.7% were female, 61.7% were non-Hispanic White, and 51.7% had employer-sponsored insurance. Additionally, 33.8% of adults were overweight or obese and 24% of adults had at least one type of respiratory condition, which included either asthma (14.7%), COPD (4.7%), or bronchitis/emphysema (11.6%).

Table 1 summarizes sociodemographic characteristics and the prevalence of self-reported physical symptoms within the past 7 days, stratified by a respiratory disease status. The overall prevalence of reporting a fever within the past 7 days was 16.2%, with significant differences observed among adults with chronic respiratory conditions when compared to those without (22.1% versus 14.4%, respectively, *p* < 0.0001). The overall prevalence of reporting chills within the past 7 days was 14.4%, with significant differences observed among adults with chronic respiratory conditions when compared to those without (19.3% versus 12.9%, respectively, *p* < 0.0001). The overall prevalence of reporting a runny or stuffy nose within the past 7 days was 14.4%, with significant differences observed among adults with chronic respiratory conditions when compared to those without (22.2% versus 12.3%, respectively, *p* < 0.0001). The overall prevalence of reporting chest congestion within the past 7 days was 15.0%, with significant differences observed among adults with chronic respiratory conditions when compared to those without (22.9% versus 12.6%, respectively, *p* < 0.0001). The overall prevalence of reporting a cough within the past 7 days was 13.6%, with significant differences observed among adults with chronic respiratory conditions when compared to those without (18.4% versus 12.1%, respectively, *p* < 0.0001). The overall prevalence of reporting a sore throat within the past 7 days was 13.6%, with significant differences observed among adults with chronic respiratory conditions when compared to those without (21.6% versus 11.2%, respectively, *p* < 0.0001). The overall prevalence of reporting a change or loss of sense of taste or smell within the past 7 days was 13.2%, with significant differences observed among adults with chronic respiratory conditions when compared to those without (18.4% versus 11.6%. respectively, *p* < 0.0001).

Table 2 summarizes the frequency of self-reported mental health symptoms within the past 7 days, stratified by a respiratory disease status. Overall, 10% of adults with chronic respiratory conditions reported feeling nervous, anxious or on edge, with significant differences compared to adults without chronic conditions (6%) (*p* < 0.001). Additionally, adults with chronic respiratory disease conditions were more likely to report feeling depressed 5–7 days per week (10% versus 6%, *p* < 0.001). Adults with chronic respiratory conditions were also more likely to report feeling lonely 5–7 days per week (11% versus 6%, *p* < 0.001). Twenty percent of adults with chronic respiratory conditions reported feeling hopeless about the future 3–7 days per week.

**Figure 1:**
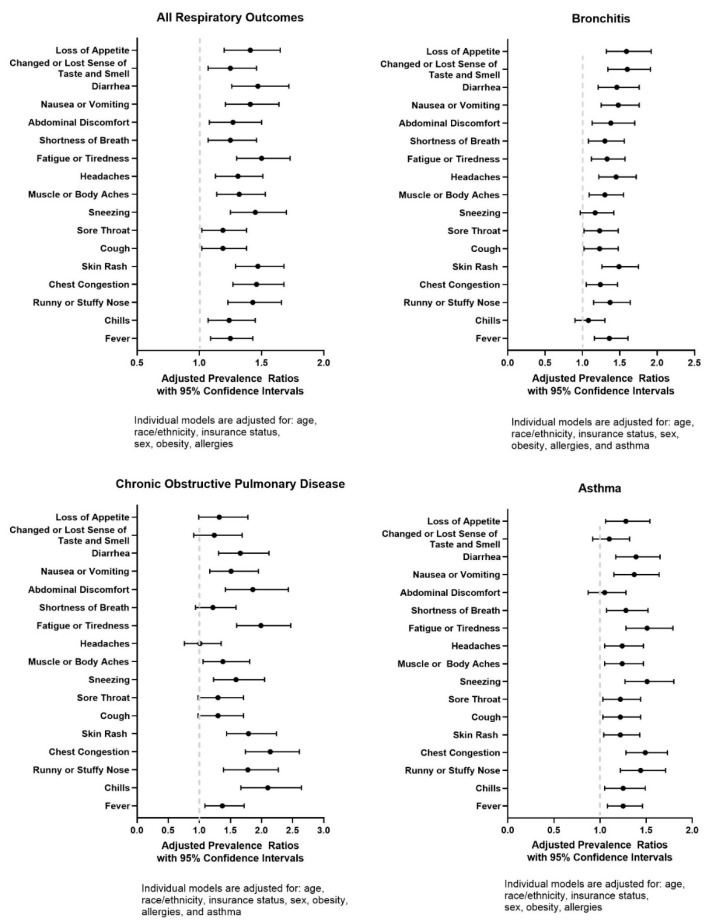
Associations of physical symptoms reported in the last 7 days among adults with respiratory conditions during the COVID-19 pandemic in the United States, derived from the COVID-19 Household Impact Survey (April–June 2020).

Figure 1 summarizes the prevalence ratios of physical symptoms by respiratory condition status after the adjustment for co-variates including age, race/ethnicity, insurance status, sex, obesity, and allergies. When we evaluate adults with any respiratory chronic condition, we find that they are more likely to report all physical symptoms when compared to adults without any history of chronic respiratory conditions. However, when we evaluate physical symptoms by specific respiratory condition, we observe differences within respiratory disease groups. Adults with asthma were not more likely to report experiencing abdominal discomfort (aPR: 1.05, 95% CI: 0.87–1.28) or changed or lost sense of taste or smell (aPR: 1.10, 95% CI: 0.92–1.32); however, they were more likely to report all other physical symptoms. Adults with chronic obstructive pulmonary diseases were more likely to report the following physical symptoms: fever (aPR: 1.37, 95% CI: 1.09–1.72), chills (aPR: 2.10, 95% CI: 1.67–2.64), runny or stuffy nose (aPR: 1.78, 95% CI: 1.39–2.27), chest congestion (aPR: 2.14, 95% CI: 1.74–2.61), skin rash (aPR: 1.44, 95% CI: 1.44–2.24), sneezing (aPR: 1.59, 95% CI: 1.23–2.05), muscle or body aches (aPR: 1.38, 95% CI: 1.06–1.81), fatigue or tiredness (aPR: 1.99, 95% CI: 1.60–2.47), abdominal discomfort (aPR: 1.86, 95% CI: 1.42–2.43), nausea or vomiting (aPR: 1.51, 95% CI: 1.17–1.95), and diarrhea (aPR: 1.31, 95% CI: 1.31–2.12). Adults with bronchitis were more likely to report all physical symptoms, excluding the following: chills (aPR: 1.08, 95% CI: 0.90–1.30) and sneezing (aPR: 1.17, 95% CI: 0.97–1.42).

**Figure 2:**
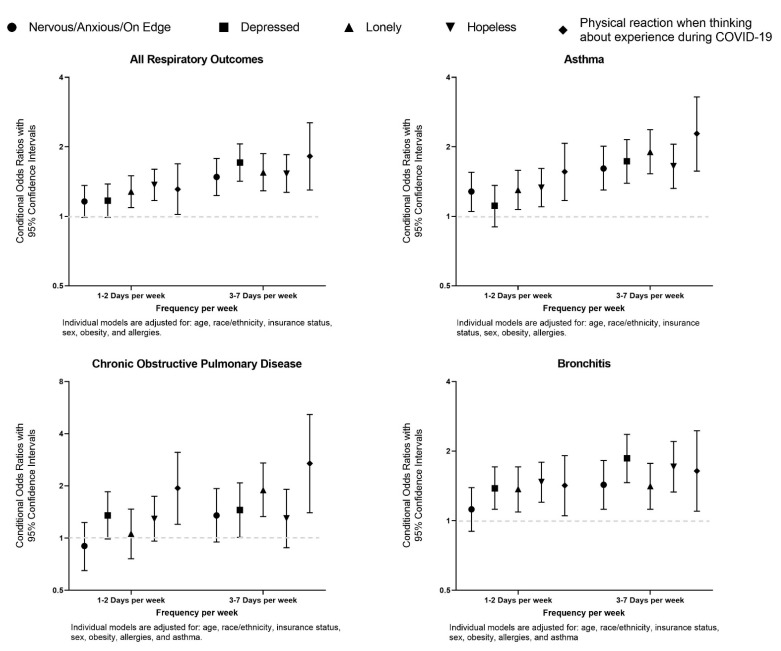
Associations of mental health symptoms reported in the last 7 days among adults with respiratory conditions during the COVID-19 pandemic in the United States, derived from COVID-19 Household Impact Survey (April–June 2020).

Figure 2 summarizes the multivariable analyses results of the odds of experiencing mental health symptoms in the past 7 days among adults with chronic respiratory conditions by any condition and each specific condition. Adults with asthma were more likely to report feeling nervous, anxious or on edge (adjusted odds ratio [aOR]: 1.61, 95% CI: 1.30–2.01), depressed (aOR: 1.73, 95% CI: 1.39–2.15), lonely (aOR: 1.90, 95% CI: 1.53–2.37), hopeless about the future (aOR: 1.65, 95% CI: 1.32–2.05), and having a physical reaction when thinking about their experiences during the COVID-19 pandemic (aOR: 2.28, 95% CI: 1.57–3.29) in the past 3–7 days per week compared to adults without asthma. Next, adults with COPD were more likely to report feeling lonely (aOR: 1.89, 95% CI: 1.33–2.71) and having a physical reaction when thinking about their experiences during the COVID-19 pandemic (aOR: 2.69, 95% CI: 1.40–5.16) compared to adults without COPD. Finally, similarly to adults with asthma, adults with bronchitis were more likely to report experiencing each mental health symptom 3–7 days per week in the past 7 days when compared to adults without bronchitis (Figure 2).

## 4. Discussion

In our study of adults living with respiratory conditions in the United States, we found that they were more likely to experience several physical health symptoms including fever, runny or stuffy nose, and fatigue or tiredness compared to the general population. We also found that adults with chronic respiratory conditions were more likely to report mental health symptoms during the COVID-19 pandemic when compared to adults without chronic respiratory conditions. These findings are consistent with other published studies documenting increased mental health symptoms reported during the COVID-19 pandemic among adults with other chronic health conditions [20]. Increased mental health symptoms reported during the COVID-19 pandemic among adults with chronic conditions may be due to the existing higher prevalence of depression and anxiety within adults with asthma and COPD [21]. While the intention of our study was not to examine change in mental health symptoms, the COVID-19 pandemic may exacerbate pre-existing mental health conditions among adults with chronic health conditions. Findings from this study may inform clinical practice and improve provider–patient communication regarding the management of mental health symptoms as the COVID-19 pandemic continues to be a pressing public health emergency in the US.

Although an increased risk of COVID-19 infection or mortality among adults with chronic respiratory conditions has not been established, individuals with respiratory conditions may fear that they are at an increased risk given early public health reports of greater COVID-19 burden among adults with chronic conditions. Our study findings suggest that the COVID-19 pandemic has had a negative impact on both the mental and physical health of adults with asthma or COPD. Although reasons for this greater burden remain unclear, the anxiety related to the perceived risk of COVID-19 due to existing symptoms may have led to this disproportionate burden. Longitudinal studies of individuals with chronic respiratory conditions should be conducted to examine the onset and persistence of mental health and physical symptoms over time. Clinical or public health messaging to inform adults with chronic respiratory conditions of the physical and mental health impacts of COVID-19, as well as of the identification of early symptoms, and resources for support may help mitigate some of the physical and mental health burdens we observed [22,23]. COVID-19 messaging interventions may be delivered within a clinical encounter for their chronic health condition, either in-person or via telehealth. Alternatively, the COVID-19 mobile health apps may be used, as our previous study has demonstrated increased acceptability of COVID-19 mHealth tools among individuals with chronic respiratory conditions.

Our study findings also demonstrated increased reports of physical health symptoms among individuals with chronic respiratory conditions. Some of the physical symptoms reported an overlap with several symptoms common to the respiratory conditions themselves including coughing, sneezing, fatigue/tiredness, and shortness of breath [24,25,26]. Adults with COPD and asthma had a particularly higher prevalence of nausea, vomiting/diarrhea, and fatigue/tiredness. COPD has also been linked to gastroesophageal reflux disease (GERD), which is known to induce feelings of nausea and vomiting, which were also more frequently reported within our analyses [27]. Previous studies of COVID-19 preventive behaviors have documented increased adherence to several preventive behaviors including social distancing, the use of face masks, and hand washing among individuals with chronic health conditions, which may help reduce COVID-19 risks [3,14]. As several studies have not observed increased COVID-19 morbidity and mortality among individuals with chronic respiratory conditions, these symptoms may be due to their pre-existing chronic condition rather than a COVID-19 diagnosis [28,29]. However, further study is warranted as adults with respiratory conditions also reported physical symptoms that are not commonly attributed to these chronic pulmonary conditions, including abdominal discomfort, skin rash, chills, fever, decreased appetite, headaches, and sore throat for all groups (COPD, asthma, and bronchitis). COVID-19-related messaging targeted towards individuals with chronic respiratory conditions may focus on helping individuals better discern potential COVID-19 symptoms from common asthma, COPD, or chronic bronchitis symptoms [30]. Mobile health tools for adults with chronic respiratory conditions may assist adults with tracking their daily symptoms to identify early onset of new symptoms [31].

Our study does have several limitations that should be considered when interpreting our study findings. Our study was based on self-reported data and we were unable to confirm the diagnosis of chronic respiratory conditions, potentially leading to misclassification or recall bias. The prevalence of asthma in our sample was similar to that found by the 2019 Behavioral Risk Factor Surveillance System and the 2019 National Health Interview Survey, indicating that our sample was reasonably nationally representative [32,33]. However, our sample’s COPD prevalence was lower than in other nationally representative samples; due to lack of data around smoking, we are unable to ascertain whether this is due to a lower risk for COPD within this population or the result of misclassification or selection bias [34]. In addition, while the COVID-19 Impact Survey provided self-reported diagnosis of respiratory conditions, the severity and management of the conditions (e.g., asthma and COPD) were unable to be ascertained from the available data. As such, it is possible that differences in the reporting of some COVID-19-related symptoms (e.g., coughing and shortness of breath) may be associated with the severity or management of the respiratory symptoms themselves rather than being true COVID-19 symptoms. Furthermore, while we were able to adjust for several key covariates within our multivariable analyses, we were unable to include smoking history, as this information was not collected through the COVID-19 Impact Survey. Additionally, individuals were not asked about their states’ COVID-19 policies or access to testing, which may have influenced their chances to report physical or mental health symptoms or receive a COVID-19 diagnosis. Finally, we were unable to compare physical and mental health symptoms in the past 7 days to self-reported data prior to the start of the pandemic. Thus, our cross-sectional study design precludes us from establishing whether differing incidences of COVID-19 symptoms among asthma, COPD, and bronchitis/emphysema patients are due to their chronic respiratory disease or severity of condition.

## 5. Conclusions

We observed that during the COVID-19 pandemic, adults with chronic respiratory conditions are more likely to experience physical and mental health symptoms compared to individuals without a history of respiratory disease. These findings have important implications given the common clinical manifestations between respiratory conditions and COVID-19. Additionally, respiratory conditions as well as COVID-19 have been independently linked to mental health symptoms. Furthermore, the COVID-19 pandemic occurred during both the spring allergy season and influenza season, and therefore presents diagnostic and treatment challenges for pulmonary physicians. Longitudinal studies are necessary to determine how to best discern between COVID-19 and chronic respiratory conditions, while also considering patients that might have both. Educational interventions via telehealth or mHealth tools may be utilized to target individuals with chronic respiratory conditions to assist with symptom tracking, early identification of COVID-19, and increased mental health support during the pandemic period.

## Figures and Tables

**Table 1:** Socio-demographic characteristics and reported symptoms among the COVID-19 Impact Survey (*n* = 10,760): a nationally representative survey of the US, stratified by respiratory condition diagnosis (April–June 2020). Column percentages (Col %) and 95% confidence intervals (CI) are included.

	Total		Adults with Chronic Respiratory Conditions		Adults without the Condition		
	Col %	95% CI	Col %	95% CI	Col %	95% CI	
Age							
18–29	20.5	19.3, 21.8	21.6	19.1, 24.3	20.2	18.8, 21.7	
30–44	25.4	24.4, 26.5	24.2	22.1, 26.4	25.8	24.6, 27.1	
45–59	24.3	23.2, 25.4	23.7	21.5, 26.0	24.5	23.2, 25.7	
60+	29.8	28.6, 30.9	30.5	28.2, 33.0	29.5	28.2, 30.8	
Sex							
Male	48.3	47.0, 49.6	40.5	37.9, 43.2	50.7	49.2, 52.2	
Female	51.7	50.4, 53.0	59.5	56.8, 62.1	49.3	47.8, 50.8	
Race/Ethnicity							
White, NH	61.7	60.3, 62.9	65.2	62.5, 67.8	60.5	59.0, 62.0	
Black, NH	11.9	11.0, 12.7	11.8	10.1, 13.7	11.9	11.0, 12.9	
Hispanic	16.5	15.5, 17.7	14.3	12.3, 16.5	17.2	16.0, 18.6	
Asian, NH	5.1	4.4, 5.8	3.5	2.5, 5.0	5.5	4.7, 6.5	
Other, NH	3.5	3.1, 3.9	3.7	2.9, 4.8	3.4	3.0, 3.9	
Education							
No HS diploma	9.8	8.8, 10.8	12.3	10.2, 14.8	9	7.9, 10.2	
HS graduate	28.2	27.0, 29.6	27.8	25.2, 30.6	28.4	26.9, 29.9	
Some college	27.7	26.7, 28.7	29.2	27.2, 31.4	27.3	26.1, 28.4	
Bachelors or above	34.3	33.1, 35.5	30.6	28.3, 33.0	35.4	34.0, 36.8	
Household Income							
<$50,000	45.8	44.5, 47.1	51.6	48.9, 54.3	44	42.5, 45.5	
$50,000–$99,999	32.1	30.9, 33.3	29.1	26.7, 31.6	33	31.6, 34.4	
≥$100,000	22.1	21.1, 23.2	19.3	17.3, 21.4	23	21.8, 24.3	
Population Density							
Rural	9.1	8.4, 9.8	9.2	7.8, 10.8	9.1	8.3, 9.9	
Suburban	18.8	17.8, 19.7	20.5	18.4, 22.7	18.2	17.2, 19.3	
Urban	72.2	71.0, 73.3	70.3	67.9, 72.7	72.7	71.4, 74.0	
Employed	49.7	48.4, 51.1	46.5	43.8, 49.2	50.7	49.2, 52.3	
Insurance Type or Health Coverage Plans							
Purchased plan	17.4	16.4, 18.5	16.9	14.9, 19.1	17.6	16.4, 18.8	
Employer-sponsored	51.7	50.3, 53.0	50.4	47.7, 53.1	52.1	50.5, 53.6	
TRICARE	4.9	4.4, 5.4	4.3	3.3, 5.5	5.1	4.5, 5.7	
Medicaid	23.5	22.4, 24.7	31.7	29.2, 34.4	21	19.8, 22.2	
Medicare	25.3	24.2, 26.4	28.1	25.8, 30.5	24.4	23.2, 25.7	
Dually eligible (Medicare and Medicaid)	9.7	9.0, 10.4	14.2	12.5, 16.1	8.3	7.5, 9.1	
VA	4.5	4.0, 5.0	5.4	4.3, 6.7	4.2	3.7, 4.8	
Indian Health Service	1.2	0.9, 1.6	2.6	1.6, 3.9	0.8	0.6, 1.1	
No insurance	8.8	8.1, 9.6	7.5	6.2, 9.0	9.2	8.3, 10.2	
Physical Symptoms Experienced in the Last 7 Days							*p*-Value
Fever	16.2	15.3, 17.2	22.1	19.9, 24.5	14.4	13.4, 15.5	<0.001
Chills	14.4	13.4, 15.4	19.3	17.2, 21.6	12.9	11.8, 14.0	<0.001
Runny or stuffy nose	14.4	13.4, 15.4	21.2	18.9, 23.6	12.3	11.3, 13.3	<0.001
Chest congestion	15	14.1, 16.0	22.9	20.6, 25.3	12.6	11.6, 13.6	<0.001
Skin rash	15.2	14.3, 16.2	23.3	21.1, 25.8	12.7	11.7, 13.8	<0.001
Cough	13.6	12.7, 14.5	18.4	16.4, 20.5	12.1	11.2, 13.2	<0.001
Sore throat	13.6	12.7, 14.5	21.6	19.4, 23.9	11.2	10.3, 12.1	<0.001
Sneezing	13.6	12.7, 14.6	20.6	18.4, 23.0	11.5	10.6, 12.5	<0.001
Muscle or body aches	13.1	12.2, 14.0	18.9	16.9, 21.0	11.3	10.4, 12.3	<0.001
Headaches	14.3	13.4, 15.2	20.6	18.4, 22.9	12.4	11.4, 13.4	<0.001
Fatigue or tiredness	13.6	12.7, 14.6	20.5	18.3, 22.8	11.5	10.5, 12.5	<0.001
Shortness of breath	12.2	11.4, 13.1	17.6	15.7, 19.8	10.6	9.7, 11.5	<0.001
Abdominal discomfort	11.1	10.3, 11.9	15.4	13.5, 17.4	9.7	8.9, 10.6	<0.001
Nausea or vomiting	12.6	11.7, 13.4	18.7	16.8, 20.8	10.6	9.8, 11.6	<0.001
Diarrhea	12.7	11.8, 13.6	19.9	17.7, 22.2	10.4	9.6, 11.4	<0.001
Changed or lost sense of taste or smell	13.2	12.3, 14.2	18.4	16.3, 20.6	11.6	10.7, 12.7	<0.001
Loss of appetite	11.5	10.7, 12.3	17.7	15.8, 19.9	9.5	8.7, 10.4	<0.001

CI: Confidence Interval; Col: Column; NH: non-Hispanic; HS: High School

**Table 2:** Self-report of mental health symptoms by respiratory disease status among COVID-19 Impact Survey respondents.

	Total		Without Chronic Respiratory Conditions		Chronic Respiratory Conditions		
	Col %	95% CI	Col %	95% CI	Col %	95% CI	*p*-value
In the past 7 days, how often have you?							
Felt nervous, anxious, or on edge							<0.001
Not at all or less than one day	61.7	60.4, 63.0	64	62.6, 65.5	54.1	51.3, 56.7	
1–2 days	21.9	20.8, 23.0	21.1	19.9, 22.3	24.5	22.2, 26.9	
3–4 days	9.6	8.8, 10.5	8.9	8.1, 9.9	11.9	10.1, 14.0	
5–7 days	6.8	6.1, 7.5	5.9	5.2, 6.8	9.6	8.1, 11.2	
Felt depressed							<0.001
Not at all or less than one day	60.9	59.6, 62.1	63.4	61.9, 64.9	52.5	49.8, 55.2	
1–2 days	22	20.9, 23.1	21.6	20.4, 22.8	23.4	21.1, 25.8	
3–4 days	9.8	9.0, 10.6	8.6	7.7, 9.5	13.7	11.9, 15.8	
5–7 days	7.4	6.7, 8.1	6.4	5.7, 7.3	10.4	8.8, 12.2	
Felt lonely							<0.001
Not at all or less than one day	60.7	59.4, 62.0	63.2	61.7, 64.7	52.5	49.7, 55.2	
1–2 days	22.9	21.8, 24.0	22.1	20.8, 23.4	25.4	23.1, 27.9	
3–4 days	9.4	8.7, 10.2	8.9	8.1, 9.9	10.9	9.4, 12.6	
5–7 days	7.1	6.4, 7.8	5.8	5.1, 6.5	11.3	9.5, 13.3	
Felt hopeless about the future							<0.001
Not at all or less than one day	60.9	59.6, 62.1	63.4	61.9, 64.8	52.7	50.0, 55.4	
1–2 days	23.4	22.4, 24.6	22.3	21.1, 23.6	27	24.7, 29.4	
3–4 days	8	7.3, 8.7	7.3	6.6, 8.1	10.2	8.6, 12.1	
5–7 days	7.7	7.0, 8.5	7	6.2, 7.9	10.1	8.6, 11.8	
Had physical reactions such as sweating, trouble breathing, nausea, or a pounding heart when thinking about their experiences during the COVID-19 pandemic							<0.001
Not at all or less than one day	90.3	89.5, 91.0	91.7	90.8, 92.5	85.6	83.6, 87.4	
1–2 days	5.9	5.3, 6.5	5.2	4.6, 5.9	7.9	6.7, 9.3	
3–4 days	2.6	2.1, 3.1	2	1.6, 2.5	4.4	3.3, 5.8	
5–7 days	1.3	1.0, 1.6	1	0.8, 1.4	2.1	1.5, 3.0	

## Data Availability

Data for these analyses are from the COVID-19 Household Impact Survey and are publicly available for download at https://www.covid-impact.org/.
